# PDGF In Aesthetics: Hype Outpacing the Evidence?

**DOI:** 10.1111/jocd.70907

**Published:** 2026-05-13

**Authors:** Miguel A. Aristizabal Torres, Michael H. Gold, Alison J. Bruce

**Affiliations:** ^1^ Department of Dermatology Mayo Clinic Jacksonville Florida USA; ^2^ Gold Skin Care Center Tennessee Clinical Research Center Nashville Tennessee USA

**Keywords:** aesthetic dermatology, aesthetic medicine, cosmetic dermatology, platelet, platelet derived growth factor (PDGF), platelet rich fibrin (PRF), platelet rich plasma (PRP)

To the Editor,

There is always hype with a new, or potentially “old” new, kid on the block. Regenerative and cosmetic treatments are not immune to current trends and the commercial buzz surrounding new biologic products is particularly notable. This may be the case with platelet derived growth factor (PDGF), a well‐known molecule, or set of molecules, that has recently seen increased use in aesthetic medicine and sparked a range of opinions about its safety, applicability, and effectiveness. The question is, as it should always be: what is the evidence?

PDGF, classically considered a derivative of platelets, is found within alpha granules in three main subforms, PDGFaa, PDGFab, and PDGFbb, classified based on their receptor‐binding profiles [1]. Upon platelet activation, PDGF is released along with other bioactive mediators such as transforming growth factor β, epidermal growth factor, and vascular endothelial growth factor. These growth factors are known to regulate key biological processes, including cell proliferation, matrix remodeling, differentiation, angiogenesis, and chemotaxis, and, along with factors from dense granules, are believed to play a central role in the clinical effects of platelet rich plasma (PRP) [2].

PDGF was one of the first growth factors to be extracted and purified, and it has long been considered one of the most potent stimulators of tissue regeneration. It is secreted not only by platelets but also by keratinocytes, fibroblasts, macrophages, and endothelial cells [3]. PDGF acts as a mitogen for fibroblasts, keratinocytes, and endothelial cells; as a chemoattractant for mesenchymal stem cells (MSCs); and promotes fibroblast proliferation and synthesis of fibronectin, collagen, proteoglycans, and hyaluronic acid, the backbone of the extracellular matrix. Receptors for PDGF are found on dermal fibroblasts, MSCs, smooth muscle cells, pericytes, glial cells, mesangial cells, osteoblasts, chondroblasts, and other cells of mesenchymal origin, allowing for spatially restricted, cell‐specific responses [4].

Its broad receptor distribution and pleiotropic cellular effects support its perceived utility in cosmetic applications. Emerging evidence further underscores its potential role in cosmetic and procedural dermatology, particularly following energy‐based procedures. In a recent study, the combination of radiofrequency microneedling with topical PDGF demonstrated superior aesthetic outcomes compared with petrolatum‐based standard care [4]. These findings may be mechanistically supported by in vitro data showing that PDGF activates NRF2‐target genes while concurrently suppressing NF‐κB–mediated inflammation in an NRF2‐dependent manner, thereby accelerating tissue recovery [5].

Theoretically, PDGF offers advantages over other regenerative modalities, such as PRP or platelet‐rich fibrin (PRF), which are subject to well‐known variability in preparation protocols. These preparations are highly operator‐ and time‐dependent, making standardization and reproducibility difficult. In contrast, purified or recombinant PDGF formulations may provide greater consistency and reproducibility, something that is urgently needed in this field [2].

Initially, topical and injectable PDGF received US Food and Drug Administration (FDA) approval for therapeutic indications such as wound healing and bone regeneration [6]. More recently, PDGF has been registered with the FDA as a topical cosmetic product, in combination with hyaluronic acid, for post‐procedural use and scar reduction [6]. This evolving regulatory status may lead some clinicians to infer that PDGF use in aesthetic medicine is both rational and safe. However, registration as a cosmetic formulation does not equate to therapeutic approval as a drug product, nor does it provide guidance on dosing, administration, or safety for injectable use.

The increasing popularity of topical PDGF formulations, both those registered with regulatory agencies and those unregistered, raises critical concerns. Notably, some products approved solely for topical cosmetic use are now being administered via injection off‐label, a practice that currently falls outside any formal regulatory safety framework and lacks standardized dosing. This misuse introduces potential safety risks, particularly in the absence of controlled clinical trials to guide appropriate dosing, administration routes, and safety monitoring. Clinicians should also be reminded that aberrant PDGF signaling has been implicated in conditions such as dermatofibrosarcoma protuberans, underscoring the potential risks of unintended cellular stimulation [7].

In conclusion, while PDGF holds promise as a regenerative agent within aesthetic medicine, its clinical use may be outpacing the available evidence. Until dosing parameters, routes of administration, and safety thresholds are established through well‐designed, controlled trials, the use of PDGF in aesthetic applications should be considered investigational rather than integrated into routine practice.

## Funding

The authors have nothing to report.

## Ethics Statement

This letter did not involve any human participants, animals, or patient data. Therefore, ethical approval was not required.

## Consent

No patients were involved in this letter; informed consent was not applicable.

## Conflicts of Interest

The authors declare no conflicts of interest.

## Data Availability

Data sharing not applicable to this article as no datasets were generated or analysed during the current study.
